# Metabolic characterization of menopause: cross-sectional and longitudinal evidence

**DOI:** 10.1186/s12916-018-1008-8

**Published:** 2018-02-06

**Authors:** Qin Wang, Diana L. Santos Ferreira, Scott M. Nelson, Naveed Sattar, Mika Ala-Korpela, Debbie A. Lawlor

**Affiliations:** 1 Computational Medicine, Faculty of Medicine, University of Oulu and Biocenter Oulu, Oulu, Finland; 2 MRC Integrative Epidemiology Unit at the University of Bristol, Oakfield House, Oakfield Grove, Bristol, BS8 2BN UK; 3 Population Health Science, Bristol Medical School, University of Bristol, Bristol, UK; 4 School of Medicine, University of Glasgow, Glasgow, UK; 5 Institute of Cardiovascular and Medical Sciences, University of Glasgow, Glasgow, UK; 6 NMR Metabolomics Laboratory, School of Pharmacy, University of Eastern Finland, Kuopio, Finland; 7 Systems Epidemiology, Baker Heart and Diabetes Institute, Melbourne, VIC Australia; 8 Department of Epidemiology and Preventive Medicine, Faculty of Medicine, Nursing and Health Sciences, School of Public Health and Preventive Medicine, The Alfred Hospital, Monash University, Melbourne, VIC Australia

**Keywords:** Metabolic profile, Reproductive status, Menopause, Post-menopausal, Cross-sectional, Longitudinal, Chronological age, Metabolomics, Circulating biomarkers

## Abstract

**Background:**

Women who experience menopause are at higher cardiometabolic risk and often display adverse changes in metabolic biomarkers compared with pre-menopausal women. It remains elusive whether the changes in cardiometabolic biomarkers during the menopausal transition are due to ovarian aging or chronological aging. Well-conducted longitudinal studies are required to determine this. The aim of this study was to explore the cross-sectional and longitudinal associations of reproductive status, defined according to the 2012 Stages of Reproductive Aging Workshop criteria, with 74 metabolic biomarkers, and establish whether any associations are independent of age-related changes.

**Methods:**

We determined cross-sectional associations of reproductive status with metabolic profiling in 3,312 UK midlife women. In a subgroup of 1,492 women who had repeat assessments after 2.5 years, we assessed how the change in reproductive status was associated with the changes in metabolic biomarkers. Metabolic profiles were measured by high-throughput quantitative nuclear magnetic resonance metabolomics. In longitudinal analyses, we compared the change in metabolic biomarkers for each reproductive-status category change to that of the reference of being pre-menopausal at both time points. As all women aged by a similar amount during follow-up, these analyses contribute to distinguishing age-related changes from those related to change in reproductive status.

**Results:**

Consistent cross-sectional and longitudinal associations of menopause with a wide range of metabolic biomarkers were observed, suggesting the transition to menopause induces multiple metabolic changes independent of chronological aging. The metabolic changes included increased concentrations of very small very low-density lipoproteins, intermediate-density lipoproteins, low-density lipoproteins (LDLs), remnant, and LDL cholesterol, and reduced LDL particle size, all toward an atherogenic lipoprotein profile. Increased inflammation was suggested via an inflammatory biomarker, glycoprotein acetyls, but not via C-reactive protein. Also, levels of glutamine and albumin increased during the transition. Most of these metabolic changes seen at the time of becoming post-menopausal remained or became slightly stronger during the post-menopausal years.

**Conclusions:**

The transition to post-menopause has effects on multiple circulating metabolic biomarkers, over and above the underlying age trajectory. The adverse changes in multiple apolipoprotein-B-containing lipoprotein subclasses and increased inflammation may underlie women’s increased cardiometabolic risk in their post-menopausal years.

**Electronic supplementary material:**

The online version of this article (10.1186/s12916-018-1008-8) contains supplementary material, which is available to authorized users.

## Background

Women in high-income countries live, on average, between 35–40% of their life in the post-menopausal state, with those in low- and middle-income countries also increasingly spending a high proportion of their lives post-menopausal [1]. Younger age at menopause is related to increased risk of osteoporosis, cardiovascular diseases (CVD), diabetes, and premature mortality, and reduced risk of ovarian cancer and breast cancer [1–7]. Similarly, a number of disease markers, such as cardiovascular risk factors [e.g., levels of low-density lipoprotein (LDL) cholesterol and apolipoprotein B}, cognitive function, and bone density, have been shown to have more adverse levels in relation to future cardiometabolic health in women who are post-menopausal compared with those who are pre-menopausal [8–12]. Assessments of biological aging, based on white cell DNA methylation, suggest that women experience an acceleration of biological aging with the onset of the menopause [13]. The differences in the risk markers between post- and pre-menopausal women could, thus, reflect the start of biological aging and disease risk related to the change in reproductive status and its associated sex hormone changes. However, the transition to post-menopause is often accompanied by the additional effects of chronological aging and midlife social adjustment [14] and in cross-sectional studies it is impossible to distinguish the effect of reproductive aging from the effect of chronological aging [11, 15].

The range of diseases related to age at menopause suggest that it may have an impact on multiple metabolic pathways. Prospective studies with repeat measurements of comprehensive metabolic profiles, together with accurate characterization of reproductive status, are required to determine this. However, to date there have been only a few prospective longitudinal studies and these have had small sample sizes or poorly defined reproductive status, or primarily explored changes in standard lipids (total cholesterol, high-density lipoprotein (HDL) cholesterol, LDL cholesterol, triglycerides), glucose, and sometimes insulin [16–19].

To study the molecular changes in response to menopausal transition and its effects independent from chronological aging, the present study investigated the cross-sectional and longitudinal associations of reproductive status, defined according to the 2012 Stages of Reproductive Aging Workshop (STRAW) criteria [20], with 74 circulating metabolic measures. These measures were primarily profiled by a high-throughput nuclear magnetic resonance (NMR) metabolomics platform [21–23], covering a wide range of metabolic pathways including lipoprotein lipids, fatty acids, amino acids, ketone bodies, and glycemic traits, which are highly relevant to cardiometabolic risk.

## Methods

**Fig. 1:**
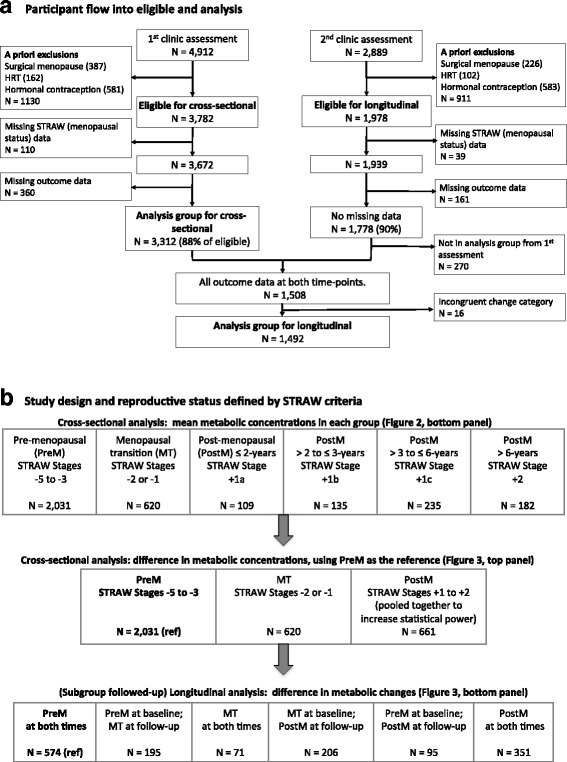
Participant flow and study design. **a** Participant flow into eligible and analysis groups. **b** Study design and reproductive status defined by STRAW criteria. HRT hormone replacement therapy, MT menopausal transition, PostM post-menopausal, PreM pre-menopausal

Data from the Avon Longitudinal Study of Parents and Children (ALSPAC) were used. Full details of the recruitment, follow-up, and data collection for these women have been reported previously and are available on the study website (http://www.alspac.bris.ac.uk) [24, 25]. ALSPAC is a prospective population-based pregnancy cohort study that recruited 14,541 pregnancies to women resident in the south west of England between 1 April 1991 and 31 December 1992. Among them, 13,761 individual women delivered at least one live birth and their children have been the main focus of a detailed follow-up since then. Approximately 18 years after their original pregnancy, a detailed assessment of the mothers was completed [25], and the current study is based on the 3,312 mothers who attended a clinic assessment between December 2008 and June 2011 [median age (interquartile range): 48 (45, 51)] and a subgroup of those women (*N* = 1,492) who attend a second follow-up assessment approximately 2.5 years later [median age (interquartile range): 51 (48, 54)]. Figure 1a illustrates the flow of women into the eligible and analysis groups. The baseline characteristics of the participating women are shown in Table 1.

**Table 1:** Baseline characteristics for ALSPAC mothers

Characteristics	Pre-menopausal (*N* = 2031)	Menopausal transition (*N* = 620)	Post-menopausal (*N* = 661)
Age (years)	46 [44–48]	50 [48–52]	54 [51–56]
Body mass index (kg/m^2^)	25 [23–29]	25 [22–29]	25 [22–29]
Height (cm)	164 [160–168]	164 [160–168]	163 [160–167]
Fat mass (kg)	25 [19–32]	24 [18–32]	25 [19–32]
Lean mass (kg)	41 [38–44]	41 [38–44]	40 [37–43]
Trunk fat mass (kg)	13 [9–18]	12 [9–17]	13 [9–17]
Systolic blood pressure (mmHg)	115 [109–124]	116 [109–125]	117 [110–127]
Diastolic blood pressure (mmHg)	70 [66–76]	71 [66–77]	72 [66–78]
Educated to university level *n*/*N* (%)^a^	301/1887 (16)	149/574 (26)	159/611 (26)
White European *n*/*N* (%)^a^	1835/1881 (98)	563/573 (98)	588/607 (97)
Lipid-lowering medication *n*/*N* (%)	29/2031 (1)	9/620 (1)	13/661 (2)

Values are presented as median [interquartile range] unless otherwise stated

^a^*n*/*N*, number with the characteristic divided by the total number in the group with no missing data on that variable

### Assessment of reproductive status (exposure)

At both clinic assessments, women were asked a range of questions regarding their menstrual cycle, including its frequency and regularity and date of last menstrual period, which enabled them to be categorized according to the STRAW criteria [20]. There are ten STRAW stages, which are grouped into three larger groups: (i) reproductive (referred to as pre-menopause in this paper), which includes categories −5 (beginning at menarche and characterized by variable to regular menstrual cycles), −4 (regular), −3b (regular) and −3a (start of subtle changes in cycle length); (ii) menopausal transition, which includes categories −2 (variable length of cycle) and −1 (categorized by episodes of amenorrhea of ≥60 days); (iii) post-menopause, which begins at the last menstrual period and includes +1a (up to the first 2 years since the last menstrual period), +1b (>2 to ≤3 years), +1c (>3 to ≤6 years), and 2 (>6 years). To increase statistical power and to reflect the precision with which the women in our study felt they could describe their menstrual cycle length and its change over time, in all analyses we combined women in all four pre-menopausal categories (i.e. −5 to −3) into one pre-menopausal category and combined those in the two menopausal transition categories (−2 and −1) into one menopausal transition category (Fig. 1b). Women were also asked about any previous hysterectomy, oophorectomy, endometrial ablation, or radio- or chemotherapy related to reproductive organs (together defined as surgical menopause), and about the use of hormone replacement and contraception. To assess naturally occurring changes across reproductive categories, we excluded a priori those women who had experienced a surgical menopause and those using hormone replacement or hormonal contraception at baseline (for both analyses) and also at follow-up (for longitudinal analyses) (Fig. 1a). The remaining women, who had completed information on STRAW data and metabolic profiles, were then classified into one of the six mutually exclusive reproductive-status groups based on the STRAW criteria (Fig. 1b, top row). None of the women were pregnant at baseline or follow-up.

### Assessment of metabolic profiling, anthropometry, and blood pressure (outcomes)

At both assessments, blood samples were taken after an overnight fast for those examined in the morning and a minimum 6 hours for those seen after 14.00. Blood samples were immediately spun and frozen at −80 °C and all assays completed for this study were undertaken within 3 years of storage and with no previous freeze/thaw cycles. A high-throughput NMR metabolomics platform was used to quantify 73 lipid and metabolite measures from stored EDTA plasma samples. The platform applies a single experimental setup, which allows for the simultaneous quantification of routine lipids, 14 lipoprotein subclasses and individual lipids transported by these particles, multiple fatty acids, glucose, various glycolysis precursors, ketone bodies, and amino acids in absolute concentration units. Details of this platform have been published previously [21, 22], and it has been widely applied in genetic and observational epidemiological studies [23, 26–34]. Highly sensitive C-reactive protein (CRP) was measured by an automated particle-enhanced immunoturbidimetric assay (Roche UK). Together, these 74 metabolic measures are defined as the primary outcomes and constitute the circulating metabolic profile.

In addition, we also analyzed the associations of reproductive status with anthropometric measures and blood pressure. Weight and height [used to calculate the body mass index (BMI)] were measured in light clothing and unshod. Weight was measured to the nearest 0.1 kg using Tanita scales; height was measured to the nearest 0.1 cm using a Harpenden stadiometer. A Lunar Prodigy dual-energy X-ray absorptiometry scan was used to measure total body fat, trunk fat, and total body lean mass. Seated blood pressure was measured with the woman at rest, her arm supported, and the correct cuff size (after measurement of arm circumference), using an Omron M6 upper arm monitor. Two measurements were taken and their mean used.

### Assessment of potential confounders

Age was recorded at each assessment. Educational attainment and ethnicity, which were considered potential confounders [17], were obtained by a questionnaire when the women were originally recruited [25]. The use of lipid-lowering medication, which affects lipid concentrations [28], was assessed by a questionnaire. Fat mass, which affects the systemic metabolic profile [33], was also considered as a potential confounder.

### Statistical analyses

The metabolic measures were scaled to standard deviation (SD) units (by subtracting the mean and dividing by the standard deviation of all women included in the baseline analysis). This scaling allows easy comparison of multiple metabolic measures with different units or with large differences in their concentration distributions.

In cross-sectional analyses, the heat map of metabolic profile across the age groups was compared to the heat map of metabolic profile across the reproductive-status groups. Baseline women were categorized into different age groups, largely defined in 1-year age categories (at the extremes of the age range, the groups were collapsed because of small numbers: 34 to 38 years and 59 to 63 years). Similarly, the same women were categorized into six reproductive-status groups according to the STRAW criteria (Fig. 1b, top row). Heat map colors illustrate the mean concentration of metabolic outcomes in each of these groups. Then, linear regression models were used to quantify the differences in the metabolic concentrations across the reproductive-status groups, using pre-menopausal women as the reference group. To increase statistical power, all four post-menopausal groups were pooled together as a single post-menopausal group (Fig. 1b, middle row).

In longitudinal analyses, a subgroup of women, who had repeated exposure and outcome measures 2.5 years later, were categorized into six groups based on their baseline and follow-up reproductive status (Fig. 1b, bottom row). Firstly, the mean difference between baseline and follow-up for each outcome in all six categories was calculated. Then, linear regression models were used to estimate the differences in mean differences between five change categories compared with those who were pre-menopausal at both baseline and 2.5 years follow-up (the reference group that primarily reflects age-related changes). To increase statistical power, these longitudinal metabolic changes associated with reproductive-stage changes were obtained by meta-analyzing results from the four groups of women (those changing from pre-menopausal to the menopausal transition, menopausal transition to 2.5 years later still in menopausal transition, menopausal transition to post-menopause, and pre- to post-menopause). Under the assumption that changing from one reproductive-status category to another is not associated with other changes that affect outcomes (beyond the confounders that we adjust for), this difference in differences is a valid means of testing causal effects with longitudinal observational data [35]. As the metabolic changes in the reference groups estimate the metabolic changes primarily due to 2.5 years of chronological aging, the results for the difference in differences can be interpreted as the extent to which the longitudinal changes deviate from the underlying age trajectory. For example, null associations illustrate that the metabolic changes that occurred during the follow-up were likely to be due only to an age-related change (and little or no impact from changing reproductive status), while pronounced associations demonstrate that the reproductive-status changes were over and above the age effect.

Due to the correlated nature of the metabolic biomarkers, over 95% of the variation in the 74 metabolic biomarkers was explained by 19 principal components. Therefore, multiple testing correction, accounting for 19 independent tests using the Bonferroni method, resulted in *P* < 0.002 (0.05/19) being denoted as statistically significant [32, 34]. All analyses were undertaken in the statistical software package R (version 3.4.0).

### Additional analyses

In the main analyses, the cross-sectional and longitudinal associations (linear regression models) were adjusted for baseline age. In additional analyses, the cross-sectional analyses were further adjusted for education, ethnicity, lipid-lowering medication, fat mass, and height, and the longitudinal analyses were additionally adjusted for baseline education, ethnicity, and 2.5-year change in lipid-lowering medication, fat mass, and height. Cross-sectional and longitudinal analyses with anthropometric and blood pressure outcomes were also undertaken. While our focus here is on metabolic profiles, these analyses allowed comparisons with previous studies on the association between reproductive status and established CVD risk factors. Lastly, as both natural menopause and surgical menopause indicate the decline of ovarian function, we examined, in those women who had been previously excluded, the cross-sectional and longitudinal associations between surgical menopause and metabolic profiles to explore whether surgical menopause had a similar association pattern to those seen for natural menopause.

## Results

Of the 3,782 eligible women attending the baseline assessment (i.e., after a priori exclusions) 3,312 (88%) had complete data on reproductive status and systemic metabolic measures. Equivalent numbers for the follow-up assessment were 1,978 and 1,778 (90%) (Fig. 1a). A total of 1,508 women had complete data at both time points. The 3,312 women with complete baseline data were included in cross-sectional analyses. The 1,492 women with complete baseline and follow-up data, and who had a valid change in reproductive status over time (16 women had a menopause status change that was not plausible, e.g., appearing to change from post-menopause to the menopause transition), were included in longitudinal analyses. Broadly similar baseline characteristics were observed for those women included in the analyses, women excluded a priori, and those excluded because of missing data (Additional file 1: Table S1).

### Metabolic heat map of chronological aging and menopause

**Fig. 2:**
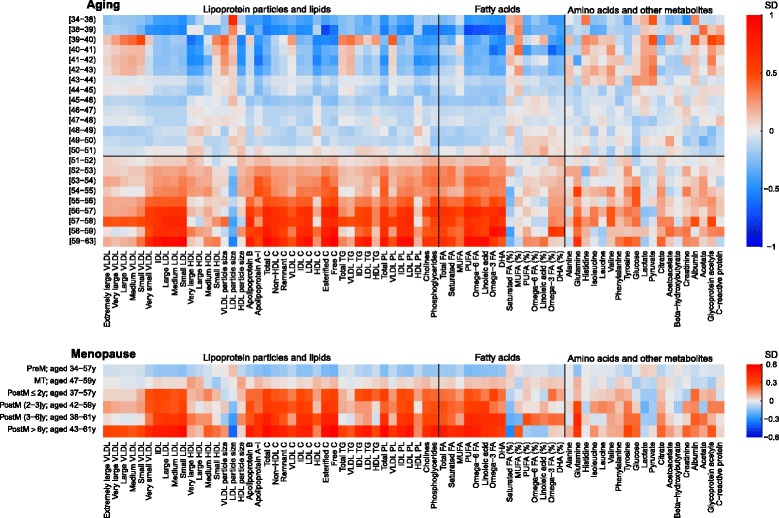
Heat map of metabolic concentrations across age groups (top panel) and across reproductive categories (bottom panel). Top panel: age groups are largely in 1-year age categories (at the extremes of age range, groups were collapsed because of small numbers: 34 to 38 years and 59 to 63 years). For each metabolic measure, mean metabolite concentrations across each age group are displayed in colors. The horizontal black line marks the typical age of natural menopause in Europe. Bottom panel: reproductive categories are defined according to the STRAW criteria. For each metabolic measure, mean metabolite concentrations across each menopausal category are displayed in colors. The age range of each reproductive category is shown on the *y*-axis. As the metabolic measures were scaled to SD units, both analyses (aging and menopause) thus used the population mean of all women at baseline as the reference value, marked as white boxes in the plots. The metabolic measures in both panels are plotted in the same order. C cholesterol, DHA docosahexaenoic acid, FA fatty acids, HDL high-density lipoprotein, IDL intermediate-density lipoprotein, MUFA monounsaturated fatty acids, PL phospholipids, PUFA polyunsaturated fatty acids, TG triglycerides, VLDL very low-density lipoprotein

Figure 2 (top panel) shows mean metabolic concentrations for women aged from the late 30s to the early 60s. Concentration of multiple lipoprotein particles, including intermediate-density lipoprotein (IDL) and LDL subclasses, and the individual lipids (triglycerides, cholesterol, and phospholipids) transported by these particles, increased with chronological age. The patterns of associations with these measures were similar, with the metabolic concentrations on average being lowest during the late 30s to mid-40s, then slightly increased during the mid-40s to early 50s, and became substantially higher from the early 50s. Similar chronological aging patterns were observed for apolipoprotein A1 and B, total cholesterol and phospholipids, absolute fatty acid concentrations, the ratio of omega-3 fatty acids and its subclass docosahexaenoic acid (DHA), as well as multiple non-lipid measures including glutamine, tyrosine, glucose, and citrate. Given the average menopausal age is 51 for those of European ancestry [36], it is plausible that the marked increases around age 50 in these metabolic profiles were associated with a reproductive-status change. Weaker increases around and after the age of menopause were seen for very low-density lipoprotein (VLDL) and HDL subclasses (except for very small VLDL), the individual lipids in these particles, and total triglycerides. LDL particle sizes became smaller with increasing age. Over and above the pattern observed around the menopausal age, we also observed an increase and then decrease in most VLDL subclasses, total and VLDL triglycerides, all branch-chain amino acids (and some of the other amino acids), lactate, pyruvate, albumin, glycoprotein acetyls, and CRP roughly around ages 39 to 43. The biological reasons or confounding factors, which could explain the increase in these metabolic measures between these ages, are, however, unclear and we have insufficient data on women in their earlier 30s to describe fully the nature of these changes.

Figure 2 (bottom panel) illustrates the mean metabolic concentrations across the reproductive groups. Multiple metabolic concentrations were substantially greater in post-menopausal compared with pre-menopausal women, with a similar pattern to that seen for the marked increase in metabolites at early 50s. Given that the reproductive groups are based on individual menstrual cycle patterns and each group has within it a large variation in age (and overlap across categories), the similar metabolic patterns suggest that the metabolic aberrations are associated with a change in reproductive status over and above chronological aging.

### Cross-sectional associations of reproductive status

**Fig. 3:**
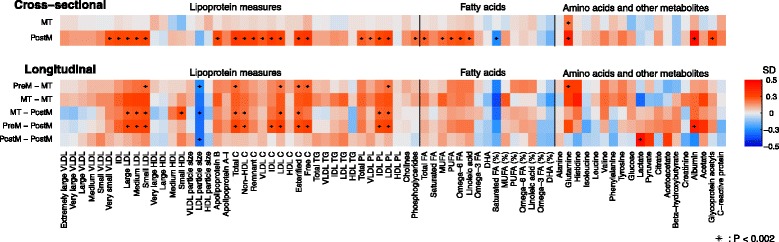
Cross-sectional (top panel) and longitudinal (bottom panel) associations of reproductive categories with 74 metabolic measures. Top panel: In the cross-sectional analysis, women were categorized into pre-menopausal, menopausal transition, and post-menopausal groups. Using pre-menopausal women as the reference group, the colors define the SD differences in each metabolite concentration between the other two groups and the reference group (Fig. 1b). Bottom panel: In the longitudinal analysis, women were categorized into six groups based on their baseline and follow-up reproductive status. Using those women who were pre-menopausal at both time points as the reference group, the colors illustrate the SD differences in the concentration change during the follow-up between the other five groups and the reference group (Fig. 1b). Cross-sectional and longitudinal associations were adjusted for baseline age. The metabolites are plotted in the same order as in Fig. 2. C cholesterol, DHA docosahexaenoic acid, FA fatty acids, HDL high-density lipoprotein, IDL intermediate-density lipoprotein, MUFA monounsaturated fatty acids, PL phospholipids, PUFA polyunsaturated fatty acids, SD standard deviation, TG triglycerides, VLDL very low-density lipoprotein

Figure 3 (top panel) shows the cross-sectional age-adjusted associations of reproductive status with 74 metabolic measures. In age-adjusted analyses, women who were post-menopausal, compared with those who were pre-menopausal, had higher concentrations of multiple lipoprotein lipids, particularly the IDL and LDL-related measures, and increased concentrations of fatty acids and several non-lipid measures. In contrast, there were only minor metabolic differences between women in the menopausal transition and those who were pre-menopausal.

### Longitudinal changes in response to reproductive-status change

Figure 3 (bottom panel) shows the longitudinal differences in mean differences for each reproductive-status change category compared with the reference category of being pre-menopausal at both time points, reflecting the amount by which outcomes changed over the follow-up period in response to a change in the reproductive status. Within each category of reproductive-status change, including the reference category of women who remained pre-menopausal at both time points, metabolite concentrations changed as all women aged 2.5 years during the follow-up (Additional file 1: Figure S1). In comparison with those women who were pre-menopausal at both time points, greater increases in IDL- and LDL-related measures, a larger decrease in LDL particle size, and greater changes in multiple fatty acid and non-lipid measures were consistently observed for the four groups of women who changed from pre-menopausal to the menopausal transition, from the menopausal transition to 2.5 years of greater exposure to the menopausal transition, from the menopausal transition to post-menopausal and from pre- to post-menopausal. These results suggest metabolic changes, over and above the underlying age trajectory, occurring during the transition period from pre- to post-menopause. By contrast, the metabolic changes for those women who were post-menopausal at both time points were less notably different to those who were pre-menopausal at both time points.

### Summary of cross-sectional and longitudinal associations of change in reproductive status

**Fig. 4:**
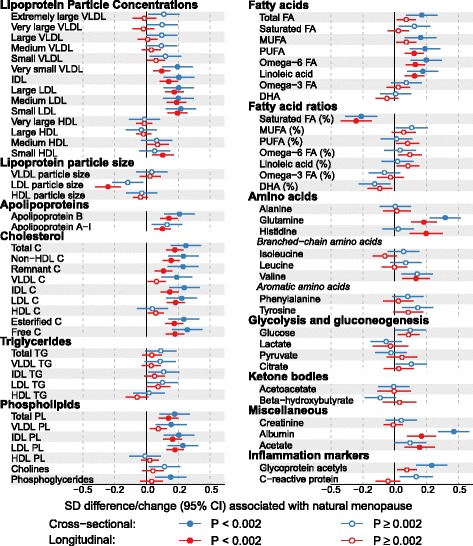
Cross-sectional (blue) and longitudinal (red) associations of natural menopause with 74 metabolic measures. The cross-sectional associations are differences in mean metabolites comparing post-menopausal to pre-menopausal women. The longitudinal associations are the differences in mean differences over time comparing four groups meta-analyzed together (pre-menopause to menopause transition, menopause transition at both times, menopause transition to post-menopause, and pre-menopause to post-menopause) to pre-menopause at both times (the reference group). The meta-analysis of the four groups, each of which at least partly represent the transition process from pre-menopausal to post-menopausal, was used to increase statistical power. Cross-sectional and longitudinal associations were adjusted for baseline age. C cholesterol, CI confidence interval, DHA docosahexaenoic acid, FA fatty acids, HDL high-density lipoprotein, IDL intermediate-density lipoprotein, MUFA monounsaturated fatty acids, PL phospholipids, PUFA polyunsaturated fatty acids, SD standard deviation, TG triglycerides, VLDL very low-density lipoprotein

Figure 4 and Additional file 1: Table S2 summarize and compare the cross-sectional and longitudinal associations of reproductive status and its change with metabolic traits. The cross-sectional results are differences in mean concentrations comparing post- to pre-menopausal women. The longitudinal association results were meta-analyzed across four groups of women (those changing from pre-menopausal to the menopausal transition, the menopausal transition to 2.5 years later still in the menopausal transition, the menopausal transition to post-menopause, and pre- to post-menopause) compared with those who were pre-menopausal at both time points. The fixed-effect meta-analysis was done to increase statistical power, given that these four groups displayed broadly similar metabolic association patterns (Fig. 3 lower panel) and that all of them represent, at least partly, the transition process from pre- to post-menopause. A meta-analysis was undertaken, as it would not have been possible to combine these women into one single change group given they changed through different categories.

Longitudinal associations of menopause were broadly similar to the cross-sectional associations (Fig. 4). Consistent associations were observed for increased concentrations of very small VLDL, IDL, and LDL particles, as well as the cholesterol and phospholipids transported in these particles, whereas there was little robust evidence from cross-sectional or longitudinal analyses in support of the associations with triglycerides. Large increases in total cholesterol, remnant cholesterol, total phospholipids, apolipoprotein B, and apolipoprotein A-1 were also observed. However, there were only weak associations with HDL-related measures. While LDL particle size decreased as women changed from pre- to post-menopause, weak changes were seen for VLDL and HDL particle sizes. Total fatty acids, omega-6 fatty acids, and the subclass of linoleic acid were increased, and the ratio of saturated fatty acids relative to total fatty acids decreased as women transitioned from pre- to post-menopause. For the non-lipid measures, large increases were observed for glutamine, histidine, valine, albumin, and acetate as women became post-menopausal. Interestingly, glycoprotein acetyls (an inflammatory marker) increased with the transition from pre- to post-menopause, whereas change in reproductive status was not robustly associated with the other inflammatory marker CRP in either longitudinal or cross-sectional analyses (the 95% confidence interval included the null in the longitudinal analyses). The magnitudes of change in these metabolic concentrations as women became post-menopausal were broadly similar (~0.2 SDs).

### Additional analyses

Cross-sectional associations of reproductive status with circulating metabolic measures remained largely unchanged when further adjusted for ethnicity, education, fat mass, height, and lipid-lowering medication (Additional file 1: Figure S2). Longitudinal associations also remained similar when adjusted for additional potential confounders, including baseline ethnicity and education and 2.5-year change in fat mass, height, and lipid-lowering medication (Additional file 1: Figure S2). Additional analyses of reproductive status with anthropometry and blood pressures were also conducted (Additional file 1: Figure S3). In cross-sectional analyses, height and lean mass were lower, and total and truncal fat mass, and diastolic blood pressure higher in post- compared to pre-menopausal women. However, with the exception of the inverse association with lean mass, these differences did not reach our multiple testing *p*-value threshold and were not replicated in longitudinal analyses. Further, there was no strong evidence for the association of reproductive-status change with BMI, waist circumference, or systolic blood pressure in either cross-sectional or longitudinal analyses. Associations of surgical menopause with multiple metabolic measures were seen in cross-sectional analysis, whereas longitudinal results were imprecisely estimated and difficult to interpret reliably due to the small number of women who underwent a surgical menopause during the follow-up (Additional file 1: Figure S4).

## Discussion

In this study of 3,312 midlife women, cross-sectional and longitudinal analyses were used to determine the relation of reproductive status with comprehensive metabolic profiles. Cross-sectional analyses showed that being post-menopausal was associated with a wide range of circulating metabolic measures, including multiple established and emerging biomarkers for type 2 diabetes, CVD, and all-cause mortality [26, 27, 37]. The difference in mean differences in the longitudinal analyses displayed broadly consistent association patterns with the cross-sectional analysis, suggesting the observed metabolic aberrations were due to the effect of reproductive-status change, over and above age-related changes. These metabolic changes were primarily toward an atherogenic lipid profile, with increased concentrations of small VLDL, IDL, and LDL subclasses, higher levels of all cholesterol measures except HDL cholesterol, increased apolipoprotein-B, and decreased LDL particle size. We also found that becoming post-menopausal resulted in a decreased proportion of saturated fatty acids, and higher concentrations of glutamine, valine, albumin, acetate, and glycoprotein acetyls. The majority of these metabolic changes appear to persist, or possibly increase slightly, over time after becoming post-menopausal.

One previous study examined cross-sectional associations of age with similar metabolic profiles to those assessed here and showed that around the average age at menopause for European women, the metabolic profiles changed more so in women than men [8], with patterns that are broadly consistent with our detailed characterization of reproductive status and longitudinal analyses. The Study of Women’s Health Across the Nation (SWAN) is the previous largest study (*N* = 1,054) to examine prospective associations of reproductive-status change with changes in established risk factors [17]. Consistent with our study, they found longitudinal increases in total cholesterol, LDL cholesterol, and apolipoprotein-B on becoming post-menopausal that were independent of chronological aging. As in our study, SWAN also found that changes in BMI, blood pressure, triglycerides, HDL cholesterol, and CRP in women’s midlife were small and/or largely expected to be related to age, rather than a change in reproductive status. Although there is little evidence supporting the change in CRP in both SWAN and our longitudinal analysis, our results suggested that the change from pre- to post-menopause increases the concentration of glycoprotein acetyls, a biomarker for low-grade chronic inflammation, which has been shown to be positively associated with diabetes, CVD, and premature mortality [26, 38–41]. Thus, becoming post-menopausal may result in increased low-grade inflammation over and above age effects. The overall consistent findings between our study and the SWAN study (which used a multilevel model analytical approach with a greater number of repeat measures) for the established risk markers also suggest that the difference in mean differences approach taken here is a valid way to assess the metabolic consequences of change in reproductive status that is independent of chronological aging.

Besides the established lipid and inflammatory markers, our results provide longitudinal evidence for a potential impact of becoming post-menopausal on lipoprotein particles, fatty acids, amino acids, and other metabolites. As women changed from pre- to post-menopause, the atherogenic lipoprotein particles, including the remnant (small VLDL + IDL) and LDL particle concentrations, increased, while LDL particle size decreased. These changes are likely to predispose post-menopausal women toward higher CVD risk. Previous studies have reported that branched-chain and aromatic amino acids are predictive of type 2 diabetes, and that their circulating levels increase in response to weight gain and increases in insulin resistance [33, 42]. Transition to post-menopause had only a weak effect on these amino acids, except for valine. These findings suggest that the adverse metabolic changes observed here are unlikely to be entirely mediated through weight gain or increased insulin resistance during the transition period, a finding consistent with our observation of little robust evidence for a relation between a change in reproductive status and a change in adiposity measurements, or a change in the longitudinal effects on metabolic traits with adjustment for both baseline and follow-up fat mass and glucose. Interestingly, the transition to post-menopause was associated with a decreased proportion of saturated fatty acids and increased concentration of glutamine and albumin, all of which are associated with lower cardiometabolic risk [26, 37, 43]. However, the casual role of these emerging biomarkers for disease risks remains unclear.

Menopause is associated with a decrease in estradiol, beginning during the transition phase (~2 years before a woman’s final menstrual period), with levels plateauing at a low value by ~2 years post-menopause, and a mirror pattern of increasing follicle stimulating hormone levels over the same period [44]. The metabolic changes observed here may, thus, reflect sex and gonadotropin hormonal changes relating to the menopausal transition. Previous studies have shown exogenous estrogen alone, or in combination with progestogens [as hormone replacement therapy (HRT) or combined hormonal contraception], is associated with a variation in lipid levels [30, 45]. Of relevance to our findings, post-menopausal women using HRT were found to have lower LDL cholesterol in comparison with those not using HRT [45]. However, these associations with exogenous hormones may not be causal, and it remains unclear to what extent HRT is associated with levels of non-lipid biomarkers, e.g. fatty acids and amino acids. Several studies have recently found exogenous hormones [30] and also reproductive-status change, including pregnancy [31] and menopause (as studied here) to be associated with changes in a wide range of circulating metabolites. Further research to understand the relationship between circulating levels of reproductive hormones at different stages of the life course and changes in comprehensive metabolic profiles would be important in giving us a better understanding of the extent to which sex hormones influence metabolism during different reproductive stages of life.

The key strengths of this study are its detailed information on reproductive status and comprehensive metabolic profiles collected on two occasions prospectively in large numbers. Our results are broadly supported by the previous large-scale cross-sectional and longitudinal studies of established risk factors [8, 17]. However, we acknowledge that replication of the associations with the emerging metabolic risk factors that we have assessed here is important. Our results suggest that the majority of the metabolic changes seen at the time of becoming post-menopausal persist over time, and for some may continue to change at a slow pace. However, the mean age of women at baseline and follow-up were 48 and 51 years, respectively, and further repeat assessments in these women would be valuable for verifying this, and to determine how long such changes may continue. Collapsing the STRAW reproductive-stage categories may have obscured some differences in our cross-sectional and longitudinal analyses. For example, in the longitudinal analyses in the SWAN study, there was an increase in conventional lipids between the early and late menopausal transition period [16, 17]. The women in our study were not able to describe confidently the change in the length of their menstrual cycle with the precision that would allow us to separate a variable length persisting for at least 7 days with a consecutive difference in length (early menopausal transition) from an interval of amenorrhea of at least 60 days (late menopausal transition) and therefore, we had to collapse these two. These two stages can also be distinguished by differences in follicle stimulating hormone levels, but we currently do not have these data. It is possible that the lack of difference between pre-menopausal women and those categorized as in the menopausal transition for most lipids in our cross-sectional analyses is because we cannot separate the early from the late menopausal transition. Furthermore, the longitudinal differences for several outcomes between those who remained in our category of menopausal transition for 2.5 years and those remaining pre-menopausal for this period may reflect that the former included a high proportion who transitioned from early to late menopausal transition status, but we are unable to determine this.

These women are currently too young to have experienced CVD events, osteoporotic fractures, or cancer in sufficient numbers for us to be able to examine the extent to which the menopause-related changes that we have observed in metabolic profiles relate to future disease outcomes. With a continued follow-up of these women that would be possible. The women in this study are largely of European origin and we cannot assume that our findings generalize to other groups.

## Conclusions

In conclusion, when women become post-menopausal, they experience changes in relation to lipoprotein metabolism, fatty acids, amino acids, and inflammation. These metabolic changes are independent of age and potentially underlie the relationship between menopause and cardiometabolic diseases. A detailed understanding of the molecular changes that occur during the menopausal transition may lead to lifestyle or therapeutic opportunities to diminish the adverse metabolic effects on women during their post-menopausal life.

## Additional file

Additional file 1: Supplementary tables and figures in support our results and findings. (DOCX 187 kb)

## Abbreviations

ALSPAC: Avon Longitudinal Study of Parents and Children
BMI: Body mass index
CRP: Highly sensitive C-reactive protein
CVD: Cardiovascular diseases
DHA: Docosahexaenoic acid
HDL: High-density lipoprotein
HRT: Hormone replacement therapy
IDL: Intermediate-density lipoprotein
LDL: Low-density lipoprotein
MUFA: Monounsaturated fatty acids
MT: Menopausal transition
NMR: Nuclear magnetic resonance
PostM: Post-menopausal
PreM: Pre-menopausal
SD: Standard deviation
STRAW: Stages of Reproductive Aging Workshop
SWAN: The Study of Women’s Health Across the Nation
VLDL: Very low-density lipoprotein
