# Supplementary material for: Metabolic characterization of menopause: cross-sectional and longitudinal evidence
Source: BMC Med. 2018 Feb 6;16:17. doi: 10.1186/s12916-018-1008-8 (PMC5800033; doi:10.1186/s12916-018-1008-8)
Supplement: Additional file 1: — Supplementary tables and figures in support our results and findings. (DOCX 187 kb) [file 12916_2018_1008_MOESM1_ESM.docx]

**Online Supplement for:**

**Metabolic characterization of menopause: cross-sectional and longitudinal evidence**

Qin Wang, Diana L Santos Ferreira, Scott M Nelson, Naveed Sattar, Mika Ala-Korpela, Debbie A Lawlor.

**Content**

**Supplementary Tables**

**Table S1.** Characteristics of women included in analyses, those *a priori* excluded and those excluded because of missing data.

**Table S2.** Mean absolute concentrations of lipoprotein, fatty acid and metabolite measures in the study population and SD concentration difference/change associated with menopause in cross-sectional and longitudinal analyses.

**Supplementary Figures**

**Figure S1.** Longitudinal change in metabolic concentrations between baseline and follow-up for the six reproduction status groups.

**Figure S2.** Additional adjustment of cross-sectional and longitudinal associations of reproductive categories with 74 metabolic measures.

**Figure S3.** Cross-sectional (blue) and longitudinal (red) associations of natural menopause with anthropometric and blood pressure outcomes.

**Figure S4.** Cross-sectional (blue) and longitudinal (red) associations of surgical menopause with 74 metabolic measures.

**Supplementary Tables**

**Table S1. Characteristics of women included in analyses, those *a priori* excluded and those excluded because of missing data.**

| Characteristics | Included in analyses  N = 3312 | A priori excluded^a^  N = 1130 | Missing data excluded  N = 470 |
| --- | --- | --- | --- |
| Age (years) | 48 [45-51] | 47 [45-50] | 48 [45-51] |
| BMI (kg/m2) | 25 [23-29] | 26 [23-30] | 26 [23-31] |
| Height (cm) | 164 [160-168] | 164 [160-168] | 163 [160-168] |
| Fat mass (kg) | 25 [19-32] | 27 [20-36] | 25 [19-36] |
| Lean mass (kg) | 41 [38-44] | 41 [38-44] | 40 [37-44] |
| Trunk fat mass (kg) | 13 [ 9-17] | 14 [10-19] | 13 [ 9-19] |
| Systolic blood pressure (mmHg) | 116 [109-124] | 118 [111-126] | 117 [110-127] |
| Diastolic blood pressure (mmHg) | 71 [66-76] | 72 [67-78] | 72 [67-78] |
| Educated to university level n/N (%)^b^ | 609/3072 (20) | 157/1036 (15) | 65/384 (17) |
| White European n/N (%)^b^ | 2986/3061 (98) | 1014/1033 (98) | 373/383 (97) |
| Lipid-lowering medication n/N (%) | 51/3312 (2) | 29/1130 (3) | 7/470 (1) |

Values are presented as median [interquartile range] unless otherwise stated.

^a^ *A priori* exclusions include those women who had a hysterectomy, oophorectomy, endometrial ablation or radio therapy to their uterus or ovaries, those taking hormone replacement therapy and those taking hormonal contraception.

^b^ n/N = number with the characteristic divided by the total number in the group with no missing data on that variable.

**Table S2.** **Mean absolute concentrations of lipoprotein, fatty acid and metabolite measures in the study population and SD concentration difference/change associated with reproduction status in cross-sectional and longitudinal analyses.**

| Metabolites  (units of absolute concentration) | Mean (SD)  of absolute concentration | SD concentration difference  associated with post- compared with pre-menopause  (**cross-sectional associations shown in Figure 4**)  Beta [95%CI]; P value | SD concentration change  associated with change from pre- to post-menopause  (**Longitudinal associations**  **shown in Figure 4**)  Beta [95%CI]; P value |
| --- | --- | --- | --- |
| **Lipoprotein Particle Concentrations** | | | |
| Extremely large VLDL (µmol/L) | 0.000067 (0.000092) | 0.14 [0.02,0.26]; P=2e-02 | -0.016 [-0.105,0.072]; P=7e-01 |
| Very large VLDL (µmol/L) | 0.00035 (0.00053) | 0.12 [0.00,0.24]; P=4e-02 | -0.024 [-0.106,0.059]; P=6e-01 |
| Large VLDL (µmol/L) | 0.0026 (0.0030) | 0.12 [0.00,0.24]; P=5e-02 | 0.0099 [-0.0632,0.0829]; P=8e-01 |
| Medium VLDL (µmol/L) | 0.011 (0.008) | 0.12 [0.00,0.24]; P=5e-02 | 0.037 [-0.034,0.108]; P=3e-01 |
| Small VLDL (µmol/L) | 0.024 (0.010) | 0.15 [0.03,0.27]; P=1e-02 | 0.076 [0.010,0.141]; P=2e-02 |
| Very small VLDL (µmol/L) | 0.034 (0.008) | 0.24 [0.13,0.36]; P=5e-05 | 0.12 [0.06,0.18]; P=2e-04 |
| IDL (µmol/L) | 0.088 (0.020) | 0.25 [0.14,0.37]; P=2e-05 | 0.17 [0.11,0.24]; P=3e-07 |
| Large LDL (µmol/L) | 0.14 (0.04) | 0.25 [0.14,0.37]; P=2e-05 | 0.22 [0.15,0.29]; P=9e-10 |
| Medium LDL (µmol/L) | 0.11 (0.03) | 0.25 [0.13,0.37]; P=3e-05 | 0.24 [0.16,0.31]; P=3e-10 |
| Small LDL (µmol/L) | 0.13 (0.03) | 0.27 [0.15,0.39]; P=5e-06 | 0.24 [0.17,0.32]; P=2e-10 |
| Very large HDL (µmol/L) | 0.47 (0.19) | -0.015 [-0.133,0.103]; P=8e-01 | -0.017 [-0.075,0.041]; P=6e-01 |
| Large HDL (µmol/L) | 1.3 (0.4) | -0.04 [-0.16,0.08]; P=5e-01 | -0.022 [-0.078,0.034]; P=4e-01 |
| Medium HDL (µmol/L) | 1.9 (0.3) | 0.08 [-0.04,0.20]; P=2e-01 | 0.087 [0.002,0.173]; P=4e-02 |
| Small HDL (µmol/L) | 4.3 (0.4) | 0.065 [-0.054,0.184]; P=3e-01 | 0.13 [0.05,0.21]; P=2e-03 |
| **Lipoprotein particle size** | | | |
| VLDL particle size (nm) | 36 (1) | 0.042 [-0.078,0.161]; P=5e-01 | 0.028 [-0.050,0.107]; P=5e-01 |
| LDL particle size (nm) | 24 (0) | -0.15 [-0.26,-0.03]; P=2e-02 | -0.30 [-0.40,-0.21]; P=5e-10 |
| HDL particle size (nm) | 10 (0) | -0.036 [-0.155,0.082]; P=5e-01 | -0.047 [-0.102,0.008]; P=9e-02 |
| **Apolipoproteins** | | | |
| Apolipoprotein B (g/L) | 0.86 (0.20) | 0.26 [0.14,0.38]; P=1e-05 | 0.18 [0.11,0.24]; P=7e-08 |
| Apolipoprotein A-I (g/L) | 1.7 (0.2) | 0.16 [0.05,0.28]; P=6e-03 | 0.13 [0.06,0.19]; P=6e-05 |
| **Cholesterol** | | | |
| Total C (mmol/L) | 4.6 (0.8) | 0.31 [0.20,0.42]; P=1e-07 | 0.22 [0.16,0.29]; P=2e-11 |
| Non-HDL C (mmol/L) | 2.9 (0.9) | 0.29 [0.17,0.41]; P=1e-06 | 0.19 [0.13,0.26]; P=2e-09 |
| Remnant C (mmol/L) | 1.4 (0.4) | 0.29 [0.17,0.40]; P=2e-06 | 0.13 [0.07,0.20]; P=6e-05 |
| VLDL C (mmol/L) | 0.67 (0.24) | 0.24 [0.12,0.36]; P=9e-05 | 0.079 [0.012,0.146]; P=2e-02 |
| IDL C (mmol/L) | 0.71 (0.18) | 0.30 [0.18,0.41]; P=5e-07 | 0.18 [0.12,0.25]; P=7e-08 |
| LDL C (mmol/L) | 1.6 (0.5) | 0.28 [0.16,0.39]; P=3e-06 | 0.23 [0.16,0.30]; P=1e-11 |
| HDL C (mmol/L) | 1.7 (0.3) | 0.047 [-0.071,0.165]; P=4e-01 | 0.074 [0.015,0.132]; P=1e-02 |
| Esterified C (mmol/L) | 3.3 (0.6) | 0.29 [0.18,0.41]; P=8e-07 | 0.22 [0.15,0.29]; P=2e-10 |
| Free C (mmol/L) | 1.4 (0.2) | 0.32 [0.20,0.44]; P=7e-08 | 0.22 [0.16,0.29]; P=2e-10 |
| **Triglycerides** | | | |
| Total TG (mmol/L) | 1.0 (0.5) | 0.11 [-0.01,0.23]; P=6e-02 | 0.042 [-0.031,0.115]; P=3e-01 |
| VLDL TG (mmol/L) | 0.63 (0.43) | 0.11 [-0.01,0.23]; P=8e-02 | 0.038 [-0.034,0.110]; P=3e-01 |
| IDL TG (mmol/L) | 0.11 (0.03) | 0.14 [0.02,0.25]; P=2e-02 | 0.064 [-0.014,0.142]; P=1e-01 |
| LDL TG (mmol/L) | 0.17 (0.06) | 0.13 [0.01,0.24]; P=4e-02 | 0.092 [0.002,0.182]; P=5e-02 |
| HDL TG (mmol/L) | 0.14 (0.03) | 0.02 [-0.10,0.14]; P=7e-01 | -0.073 [-0.158,0.011]; P=9e-02 |
| **Phospholipids** | | | |
| Total PL (mmol/L) | 3.2 (0.4) | 0.22 [0.11,0.34]; P=2e-04 | 0.17 [0.10,0.25]; P=3e-06 |
| VLDL PL (mmol/L) | 0.40 (0.16) | 0.19 [0.08,0.31]; P=1e-03 | 0.093 [0.029,0.157]; P=5e-03 |
| IDL PL (mmol/L) | 0.30 (0.07) | 0.25 [0.14,0.37]; P=2e-05 | 0.21 [0.14,0.28]; P=9e-09 |
| LDL PL (mmol/L) | 0.64 (0.14) | 0.29 [0.17,0.40]; P=1e-06 | 0.22 [0.16,0.29]; P=3e-11 |
| HDL PL (mmol/L) | 1.9 (0.3) | -0.012 [-0.130,0.107]; P=8e-01 | 0.027 [-0.039,0.092]; P=4e-01 |
| Cholines (mmol/L) | 2.0 (0.3) | 0.14 [0.02,0.26]; P=2e-02 | 0.051 [-0.028,0.131]; P=2e-01 |
| Phosphoglycerides (mmol/L) | 1.9 (0.3) | 0.19 [0.07,0.31]; P=2e-03 | 0.041 [-0.043,0.125]; P=3e-01 |
| **Fatty acids** | | | |
| Total FA (mmol/L) | 11 (2) | 0.21 [0.09,0.33]; P=5e-04 | 0.091 [0.021,0.161]; P=1e-02 |
| Saturated FA (mmol/L) | 4.1 (0.8) | 0.15 [0.04,0.27]; P=1e-02 | 0.029 [-0.050,0.109]; P=5e-01 |
| MUFA (mmol/L) | 2.8 (0.8) | 0.20 [0.08,0.32]; P=9e-04 | 0.091 [0.017,0.166]; P=2e-02 |
| PUFA (mmol/L) | 4.2 (0.7) | 0.24 [0.12,0.35]; P=7e-05 | 0.15 [0.08,0.22]; P=2e-05 |
| Omega-6 FA (mmol/L) | 3.7 (0.6) | 0.25 [0.13,0.37]; P=4e-05 | 0.16 [0.09,0.24]; P=1e-05 |
| Linoleic acid (mmol/L) | 2.9 (0.5) | 0.22 [0.10,0.34]; P=2e-04 | 0.16 [0.08,0.23]; P=4e-05 |
| Omega-3 FA (mmol/L) | 0.44 (0.12) | 0.092 [-0.025,0.209]; P=1e-01 | 0.028 [-0.061,0.116]; P=5e-01 |
| DHA (mmol/L) | 0.15 (0.05) | 0.0071 [-0.1105,0.1248]; P=9e-01 | -0.061 [-0.146,0.024]; P=2e-01 |
| **Fatty acid ratios** | | | |
| Saturated FA (%) | 37 (2) | -0.26 [-0.38,-0.14]; P=2e-05 | -0.30 [-0.42,-0.18]; P=5e-07 |
| MUFA (%) | 25 (3) | 0.13 [0.01,0.25]; P=3e-02 | 0.071 [-0.020,0.161]; P=1e-01 |
| PUFA (%) | 38 (3) | 0.015 [-0.107,0.136]; P=8e-01 | 0.10 [0.01,0.19]; P=2e-02 |
| Omega-6 FA (%) | 34 (3) | 0.042 [-0.080,0.164]; P=5e-01 | 0.12 [0.03,0.21]; P=8e-03 |
| Linoleic acid (%) | 27 (3) | 0.022 [-0.099,0.144]; P=7e-01 | 0.10 [0.02,0.19]; P=2e-02 |
| Omega-3 FA (%) | 4.0 (0.8) | -0.081 [-0.201,0.040]; P=2e-01 | -0.033 [-0.135,0.069]; P=5e-01 |
| DHA (%) | 1.3 (0.4) | -0.16 [-0.28,-0.04]; P=1e-02 | -0.12 [-0.21,-0.02]; P=1e-02 |
| **Amino acids** | | | |
| Alanine (mmol/L) | 0.25 (0.06) | 0.0049 [-0.1145,0.1243]; P=9e-01 | 0.017 [-0.087,0.122]; P=7e-01 |
| Glutamine (mmol/L) | 0.48 (0.06) | 0.40 [0.28,0.51]; P=3e-11 | 0.23 [0.13,0.33]; P=4e-06 |
| *Branched-chain amino acids* | | | |
| Isoleucine (mmol/L) | 0.034 (0.013) | 0.07 [-0.05,0.19]; P=2e-01 | -0.077 [-0.167,0.014]; P=1e-01 |
| Leucine (mmol/L) | 0.05 (0.01) | 0.087 [-0.032,0.206]; P=2e-01 | -0.0013 [-0.0973,0.0946]; P=1e+0 |
| Valine (mmol/L) | 0.14 (0.03) | 0.18 [0.06,0.30]; P=4e-03 | 0.17 [0.06,0.27]; P=2e-03 |
| *Aromatic amino acids* | | | |
| Phenylalanine (mmol/L) | 0.044 (0.006) | 0.10 [-0.02,0.22]; P=9e-02 | 0.029 [-0.086,0.144]; P=6e-01 |
| Tyrosine (mmol/L) | 0.054 (0.012) | 0.18 [0.06,0.30]; P=3e-03 | 0.11 [-0.01,0.22]; P=6e-02 |
| Histidine (mmol/L) | 0.056 (0.012) | 0.027 [-0.092,0.147]; P=7e-01 | 0.25 [0.12,0.37]; P=1e-04 |
| **Glycolysis and gluconeogenesis** | | | |
| Glucose (mmol/L) | 4.5 (0.7) | 0.12 [0.00,0.24]; P=4e-02 | 0.11 [0.03,0.19]; P=1e-02 |
| Lactate (mmol/L) | 0.79 (0.33) | -0.067 [-0.186,0.052]; P=3e-01 | -0.034 [-0.169,0.102]; P=6e-01 |
| Pyruvate (mmol/L) | 0.088 (0.029) | -0.03 [-0.15,0.09]; P=6e-01 | 0.057 [-0.059,0.174]; P=3e-01 |
| Citrate (mmol/L) | 0.095 (0.027) | 0.13 [0.01,0.25]; P=3e-02 | 0.032 [-0.082,0.146]; P=6e-01 |
| **Ketone bodies** | | | |
| Acetoacetate (mmol/L) | 0.033 (0.025) | -0.0072 [-0.1266,0.1123]; P=9e-01 | -0.0046 [-0.1309,0.1216]; P=9e-01 |
| Beta-hydroxybutyrate (mmol/L) | 0.11 (0.11) | -0.11 [-0.23,0.01]; P=6e-02 | 0.038 [-0.091,0.167]; P=6e-01 |
| **Miscellaneous** | | | |
| Creatinine (mmol/L) | 0.062 (0.010) | 0.052 [-0.067,0.171]; P=4e-01 | -0.012 [-0.083,0.059]; P=7e-01 |
| Albumin (cu) | 0.091 (0.004) | 0.46 [0.35,0.58]; P=2e-14 | 0.21 [0.10,0.32]; P=2e-04 |
| Acetate (mmol/L) | 0.064 (0.025) | 0.12 [0.00,0.24]; P=5e-02 | 0.20 [0.08,0.31]; P=8e-04 |
| **Inflammation markers** | | | |
| Glycoprotein acetyls (mmol/L) | 1.2 (0.2) | 0.29 [0.17,0.41]; P=2e-06 | 0.096 [0.023,0.169]; P=1e-02 |
| C-reactive protein (mg/L) | 2.0 (3.5) | 0.17 [0.04,0.29]; P=8e-03 | -0.053 [-0.145,0.040]; P=3e-01 |

All the 73 lipoprotein, lipid and metabolite measures were quantified using the same high‑throughput serum NMR metabolomics platform. The 14 lipoprotein subclass sizes were defined as follows: extremely large VLDL with particle diameters from 75 nm upwards and a possible contribution of chylomicrons, five VLDL subclasses (average particle diameters of 64.0 nm, 53.6 nm, 44.5 nm, 36.8 nm, and 31.3 nm), IDL (28.6 nm), three LDL subclasses (25.5 nm, 23.0 nm, and 18.7 nm), and four HDL subclasses (14.3 nm, 12.1 nm, 10.9 nm, and 8.7 nm). The mean size for VLDL, LDL and HDL particles was calculated by weighting the corresponding subclass diameters with their particle concentrations.

Remnant cholesterol was defined as VLDL-cholesterol + IDL-cholesterol, which is equivalent to total‑cholesterol - HDL-cholesterol - LDL-cholesterol.

cu: standardized concentration units.

**Supplementary figures**

**Figure S1. Longitudinal change in metabolic concentrations between baseline and follow-up for the six reproduction status groups.** For each metabolic measure, the absolute concentration changes between baseline and follow-up were scaled to baseline SD-units. The mean of the changes across the six menopausal groups were then calculated and visualized.

**Figure S2. Additional adjustment of cross-sectional and longitudinal associations of reproductive categories with 74 metabolic measures.** The cross-sectional associations were adjusted for age, education, ethnicity, total fat mass, height, and lipid-lowering medication. Longitudinal associations were adjusted for baseline age, education, ethnicity and the change of total fat mass, height and lipid-lowering medication during the follow-up.

All of the confounders that we assessed were a priori considered common causes for menopausal status at a given age and also for metabolic concentrations. For example, maternal educational attainment is a marker of socioeconomic position that has been shown to be robustly related to lifestyle, including diet, physical activity and smoking etc., which we considered would plausibly influence menopausal status and metabolite levels. However, our sensitivity analyses suggested that the results remained similar when further adjusted for ethnicity, education, fat mass, height and lipid-lowering medication, suggesting that the associations we have identified are not markedly biased by residual confounding.

**Figure S3. Cross-sectional (blue) and longitudinal (red) associations of natural menopause with anthropometric and blood pressure analyses.** The cross-sectional associations are differences in mean metabolites comparing post-menopausal to pre-menopausal women. The longitudinal associations are the differences in mean differences over time comparing four groups meta-analyzed together (PreM - MT, MT - MT, MT - PostM, PreM - PostM) to preM-preM (reference group); these represent the transition period from pre-menopausal to post-menopausal. Cross-sectional and longitudinal associations were adjusted for baseline age.

**Figure S4. Cross-sectional (blue) and longitudinal (red) associations of surgical menopause with 74 metabolic measures.** In cross-sectional analysis, 348 women with surgical menopause were compared to 2031 pre-menopausal women. In longitudinal analysis, the change in metabolic concentrations for those women who were at pre-menopausal at baseline but then had surgical menopause at follow-up (N = 23), were compared to those women who were at pre-menopausal at both time-points (N = 574). Both cross-sectional and longitudinal associations were adjusted for baseline age. Women with surgical menopause were defined as those who experienced hysterectomy, oophorectomy, endometrial ablation or radio- or chemotherapy.
